# Estimating an individual-level deprivation index for HIV/HCV coinfected persons in Canada

**DOI:** 10.1371/journal.pone.0249836

**Published:** 2021-04-19

**Authors:** Adam Palayew, Alexandra M. Schmidt, Sahar Saeed, Curtis L. Cooper, Alexander Wong, Valérie Martel-Laferrière, Sharon Walmsley, Joseph Cox, Marina B. Klein, for the Canadian Coinfection Cohort Study Investigators

**Affiliations:** 1 Epidemiology, Biostatistics & Occupational Health, McGill University, Montreal, Quebec, Canada; 2 Ottawa Hospital Research Institute, Ottawa, Ontario, Canada; 3 Canadian Institutes of Health Research, Canadian HIV Trials Network, Vancouver, British Columbia, Canada; 4 Department of Medicine, University of Saskatchewan, Regina, Saskatchewan, Canada; 5 Department of Microbiology and Infectious Diseases, Centre de recherche du Centre hospitalier de l’Université de Montréal, Montreal, Quebec, Canada; 6 University Health Network, University of Toronto, Toronto, Canada; 7 Division of Infectious Diseases and Chronic Viral Illness Service, Department of Medicine, McGill University Health Centre, Montreal, Quebec, Canada; University of Arkansas for Medical Sciences, UNITED STATES

## Abstract

**Background:**

HIV-HCV coinfected individuals are often more deprived than the general population. However, deprivation is difficult to measure, often relying on aggregate data which does not capture individual heterogeneity. We developed an individual-level deprivation index for HIV-HCV co-infected persons that encapsulated social, material, and lifestyle factors.

**Methods:**

We estimated an individual-level deprivation index with data from the Canadian Coinfection Cohort, a national prospective cohort study. We used a predetermined process to select 9 out of 19 dichotomous variables at baseline visit to include in the deprivation model: income >$1500/month; education >high school; employment; identifying as gay or bisexual; Indigenous status; injection drug use in last 6 months; injection drug use ever; past incarceration, and past psychiatric hospitalization. We fitted an item response theory model with: severity parameters (how likely an item was reported), discriminatory parameters, (how well a variable distinguished index levels), and an individual parameter (the index). We considered two models: a simple one with no provincial variation and a hierarchical model by province. The Widely Applicable Information Criterion (WAIC) was used to compare the fitted models. To showcase a potential utility of the proposed index, we evaluated with logistic regression the association of the index with non-attendance to a second clinic visit (as a proxy for disengagement) and using WAIC compared it to a model containing all the individual parameters that compose the index as covariates.

**Results:**

We analyzed 1547 complete cases of 1842 enrolled participants. According to the WAIC the hierarchical model provided a better fit when compared to the model that does not consider the individual’s province. Values of the index were similarly distributed across the provinces. Overall, past incarceration, education, and unemployment had the highest discriminatory parameters. However, in each province different components of the index were associated with being deprived reflecting local epidemiology. For example, Saskatchewan had the highest severity parameter for Indigenous status while Quebec the lowest. For the secondary analysis, 457 (30%) failed to attend a second visit. A one-unit increase in the index was associated with 17% increased odds (95% credible interval, 2% to 34%) of not attending a second visit. The model with just the index performed better than the model with all the components as covariates in terms of WAIC.

**Conclusion:**

We estimated an individual-level deprivation index in the Canadian Coinfection cohort. The index identified deprivation profiles across different provinces. This index and the methodology used may be useful in studying health and treatment outcomes that are influenced by social disparities in co-infected Canadians. The methodological approach described can be used in other studies with similar characteristics.

## Introduction

HIV/HCV co-infected individuals are often marginalized and of lower socio-economic status [1]. Routes of HIV and HCV transmission overlap and are exacerbated by up-stream social determinants of health such as injection drug use [2]. The concept of deprivation was defined by Townsend in 1987 as “*a state of observable and demonstrable disadvantage relative to the local community or the wider society or nation to which that individual*, *family*, *or group belongs*” [3]. However, deprivation is a latent construct and cannot be directly measured nor estimated using a single variable. Therefore, we must use several variables but these variables may be highly collinear. To measure deprivation, studies aim to isolate the common variation inherent to several variables and summarize it with numerical scores. The most common measure of deprivation in Canada is the Pampalon Index which was developed in Quebec [4]. The Pampalon index is a 6-item index based on an aggregate of a census area, described by the proportion of the population that are: employed, with high school education or more, separated or widowed or divorced, single parent families, live alone, and the average household income for a given census area [5]. The Pampalon index has been used to measure deprivation in other provinces in Canada, in HIV mono-infected, HCV mono-infected, and HIV-HCV co-infected individuals in British Columbia with administrative data [5–7].

While the Pampalon index is useful in describing the deprivation of a census area, it is limited in its ability to describe deprivation among individuals in observational studies. First, it is an area-based index. Area-based indices assume that deprivation is homogeneous across the entire geographic area of measurement, which can lead to a loss of individual-level information by grouping people together who have different individual needs [8, 9]. Second, the Pampalon index was primarily designed and validated to measure deprivation in the general population, which may not be applicable to more marginalized populations such as people living with HIV and HCV [10]. Variables that help provide good differentiation in the Pampalon index such as the proportion of people who are widowed, separated or divorced may be useful to distinguish between members of the general population, but may not differentiate well for more marginalized populations where marriage is less common. On the other hand, characteristics such as injection drug use and incarceration history may be more useful in distinguishing levels of deprivation within HIV-HCV co-infected populations.

The primary aim of this study was to develop and estimate an individual-level model-based deprivation index that considers social, material, and lifestyle variables for members of the Canadian co-infection cohort (CCC) using an item response theory (IRT) model. Secondly, we explored the differences in the index among four regions in Canada, and how the parameters of the index varied by the different regions. Additionally, to provide an example of how the proposed index could be used, we looked at an outcome potentially impacted by deprivation and compared the fitted model with only the index as a covariate against the model that had all the variables that make up the index included as covariates.

## Methods

### Study setting and participants

The CCC is a publicly funded open prospective cohort of approximately 2000 HIV/HCV co-infected individuals recruited from 18 centers across Canada. Since 2003, HIV+ adults with evidence of HCV infection (antibody positive) have been eligible to participate. Data on socio-demographic, behavioral, and clinical characteristics and laboratory data are collected bi-annually. Details on HIV and HCV treatments/responses, clinical diagnoses, and deaths are collected using standardized case report forms [11]. The data we used for this study spanned all first visits for the CCC from 2003 to the third quarter of 2018.

### Development of the index

Variable pool development: We considered 19 variables potentially associated with material and social deprivation for inclusion in the model. For all analyses, all the variables were dichotomized. For our model, it was important that all the variables had a clear hierarchy, since the model assumes that all levels of every variable are monotonically increasing [12]. The variables considered for inclusion fell into two categories: 1) the 6 Pampalon variables and 2) social and behavioral variables associated with deprivation in the literature.

#### Pampalon variables

We adapted five out of six variables used to estimate the Pampalon index. The variables were: high school education or less, living alone (yes/no), relationship status (single/married), employed (yes/no), and income dichotomized into equal to or more than $1500 a month and less than $1500 (based on low income threshold for a single person in Canada in 2008, which was the last year an income poverty line was used in Canada) [13]. The proportion of single parent homes was the one variable from the Pampalon index that did not have corresponding individual level data collected by the CCC.

#### Variables selected from the literature

These variables were selected after performing a literature search in Medline, EMBASE, and PsychInfo focusing on articles reporting on social/behavioral factors associated with HIV/HCV co-infection. Variables identified from the literature were then matched to individual level variables available in our cohort, the reference group for these studies were either HIV or HCV mono-infected [7, 14–19]. We considered 14 dichotomous (yes/no) variables, which included: identifying as gay or bisexual, vulnerably housed (defined as being homeless or living in a shelter), snorted drugs in the last 6 months, injected drugs ever, injected drugs in last 6 months, ever incarcerated, sex client in the last 6 months, sex work in the last 6 months, sex client ever, sex work ever, sexually transmitted infection in the last 6 months, depression, hospitalized at a psychiatric institution, and diagnosed with schizophrenia.

#### Variables selection process

Variables were selected for inclusion into the final model using a pre-defined variable selection process. For each group of variables, all possible combinations of two-way *χ*^2^ significance tests (set at an *α* of 0.05), and the grouping between variables in output from multiple correspondence analysis (MCA) were evaluated [20].

### Proposed model

We fit an IRT model with all complete cases of the final selected variables [21]. We used an IRT model because it allowed us to estimate an individual-level deprivation index for every member of our cohort and evaluate the relative importance of every item in determining an individual’s score. We fit two models: one that ignored groupings and another that grouped observations hierarchically based on an individual’s province of recruitment. The purpose of allowing the items of the model to vary by province is that there are known differences in sociodemographic factors in participants residing from different provinces (such as the prevalence of Indigenous populations, income, drug use) that could influence an individual’s level of deprivation [22].

Our proposed model follows Quinn 2004 [23]. Let *x*_*ijk*_ be the response to the j-th item for person *i* from province *k*. The hierarchical IRT model assumes there is a latent continuous variable such that if $x_{ijk}=0\;then{\;x}_{ijk}^{*}\in \left(-\infty ,0\right)$ and if *x*_*ijk*_ = 1 then $x_{ijk}^{*}\in \left[0\right.,\left.\infty \right)$, with $x_{ijk}^{*}=\alpha_{jk}+\beta_{jk}\theta_{ik}$, with *∊*_*ijk*_ ~ *N*(0,1). The parameter *α*_*jk*_ is the response parameter and the parameter *β*_*jk*_ is the discrimination parameter. The parameter *θ*_*ik*_ is the index associated with subject *i*, and is what we are considering as our individual-level deprivation index. Note that we allow *α*_*jk*_ and *β*_*jk*_ to vary with the province, *a priori*. This allows the data to drive the inference procedure and point out if there are differences at the provincial level in the way the variables influence the estimation of the index.

#### Item characteristic curves

An item characteristic curve (ICC) is used to evaluate the probability of responding yes to a given item based on an individual’s latent score [12]. The ICC is defined for the two-parameter IRT model as $P\left(\theta_{ik}\right)=\frac{1}{1+e^{-\beta_{jk}\left(\theta_{ik}-\alpha_{jk}\right)}}$. The response parameter (*α*_*jk*_) is the location of the inflection point of an ICC, and the discriminatory parameter (*β*_*jk*_) is the slope of an ICC around its point of inflection [24]. This allowed us to examine how the different items discriminate as a function of the subject parameter (*θ*_*ik*_), which is our index. We further examined how the ICCs varied in the different provinces.

#### Model selection

We compared the model that considered provincial grouping (assuming α_jk_ and β_jk_ varied by province) to the one that did not (assuming α_j_ and β_j_ common across provinces). We calculated the widely-applicable information criteria (WAIC) to evaluate which model, between the fitted ones, best fits the data [25]. The model with the lowest WAIC was chosen as the index. A difference of 5 in WAIC was considered to be substantial [26].

#### Association between the index and not attending a second visit

Secondary to estimating the index, we examined the association of the deprivation index at baseline and not attending a second cohort visit (scheduled every 6 months)—as a proxy for disengagement in care. This was to provide an example of a potential use of the estimated index, but not to accurately model the probability of attending a second cohort visit. We fitted a model that considered only our estimated index as a covariate, as it summarizes through a single number the characteristics of the individual, and another model that considered as covariates each of the variables that are considered in the estimation of the index. All models were fitted using a logistic regression. The WAIC was used to determine the model that best fit the data. We chose to use logistic regression because the time between the baseline and when the outcome is assessed is fixed for all individuals obviating the need for time to event analysis. This analysis was not to make predictions for future outcomes in the cohort.

#### Statistical analyses

All analyses, graphics, and proposed model fits were done following the Bayesian paradigm using the software R [27] and the Stan programming language [28]. Functions from the package RStan, provide samples from the posterior distribution of the parameters in the model via Hamiltonian Monte Carlo with a No U-Turn Sampler [29]. The 95% posterior credible intervals for each parameter is presented after 5000 iterations with a thinning interval of 1 and 2500 burn-in iterations for 4 chains. Convergence of the model was determined through the examination of trace plots and R-hat values. The WAIC for each model was calculated using the WAIC function from the loo Package [30].

### Availability of data and materials

A fictitious sample generated based on the posterior summaries of the parameters is also available, but the original data is not publicly available as it requires approval from the CCC to be shared.

### Ethics statement

The study has been approved by research ethics boards at each of the participating institutions as follows: community advisory committee of the CIHR-Canadian HIV Trials Network, the Biomedical B Research Ethics Board of the McGill University Health Centre (2006–1875 and BMB-06-006t), the UBC-Providence Health Care Research Ethics Board (H16-02853), the Institutional Review Board Services, Regina Qu’Appelle Health Region Research Ethics Board (REB-14-70), the Conjoint Health Research Ethics Board of the University of Calgary (REB15-0848 REN5), the Nova Scotia Health Research Ethics Board (1022541), the Windsor Regional Hospital Research Ethics Board (07-122-17), the Veritas Independent Review Board (7035–11:502-04-2020), the Hamilton Integrated Research Ethics Board (06–397), the Comité d’éthique de la recherche du CHUM (2003–1582, SL 03.008-BSP), the Comité d’éthique de la recherche du CHU de Québec-Université Laval (2012–876, C11-12-153), the Sunnybrook Health Sciences Centre Research Ethics Board (252–2008), the Research Ethics Board of Health Sciences North (605), the University Health Network Research Ethics Board (06-0629-BE), the Ottawa Health Science Network Research Ethics Board (2007229-01H) and the Biomedical Research Ethics Board (12–178). The study was conducted according to the Declaration of Helsinki. Patient records/information was anonymized and de-identified prior to analysis. All patients gave written informed consent before undergoing an initial evaluation and were followed at study visits every six months.

## Results

Overall, 1842 individuals were enrolled in the CCC between its inception and the third quarter of 2018. At cohort entry, the median age was 54 years, 28% were female, 24% Indigenous and 82% reported ever using injection drugs. The median CD4 count was 410 cells/μL, 31 individuals had HIV RNA greater than or equal to 50 copies/mL, and 91% were receiving antiretroviral therapy; 46% had ever received HCV treatment.

### Variable selection

We initially considered 19 variables (Table 1), 5 from the Pampalon group and 14 from the literature group. After applying the selection process, 9 variables, which are stared in Table 1, were selected and used to fit the final IRT model. The output for the *χ*^2^ tests and the MCA for both groups of variables are presented in S1 File along with the final list of candidate variables that were considered for inclusion into the index. We analyzed 1574 complete cases of a possible 1842 enrolled participants for the 9 variables included into the model. Participants from Nova Scotia (n = 13) and Alberta (n = 47) were excluded since there were not enough individuals to evaluate the group level parameters in the hierarchical model.

**Table 1:** Characteristics of the participants at baseline according to variables that were considered for inclusion into the model stratified by province.

	Quebec	Ontario	British Columbia	Saskatchewan
n	613	409	563	197
Education level (%)*				
High school diploma or less	461 (75)	255 (62)	400 (71)	179 (90)
More than high school	149 (24)	153 (37)	152 (27)	17 (8)
Missing	3 (0.5)	1 (0.2)	11 (2)	1 (1)
Vulnerably housed (%)				
Housed	493 (80)	391 (96)	498 (89)	180 (91)
Vulnerably housed	107 (18)	18 (4)	59 (11)	17 (9)
Missing	13 (2)	0 (0)	6 (1)	0 (0)
Living alone (%)				
Not alone	241 (39)	209 (51)	221 (39)	128 (65)
Alone	359 (59)	200 (49)	336 (60)	69 (35)
Missing	13 (2)	0 (0)	6 (1)	0 (0)
Income (%)*				
< = $1500 CAD/month	508 (83)	265 (65)	426 (76)	158 (80)
> $1500 CAD/month	99 (16)	139 (34)	129 (23)	29 (15)
Missing	6 (1)	5 (1)	8 (1)	10 (5)
Marital Status (%)				
Married	76 (12)	98 (24)	113 (20)	52 (26)
Not Married	520 (85)	311 (76)	439 (78)	145 (74)
Missing	17 (3)	0 (0)	11 (2)	0 (0)
Employment (%)*				
Unemployed	486 (79)	319 (78)	444 (79)	162 (82)
Employed	126 (21)	89 (22)	111 (20)	34 (17)
Missing	1 (0)	1 (0)	8 (1)	1 (1)
Non-GBMSM (%)*				
Identifying as gay or/bisexual	151 (25)	154 (38)	103 (18)	7 (4)
Identifying as heterosexual	456 (74)	254 (62)	445 (79)	190 (96)
Missing	6 (1)	1 (0)	15 (3)	0 (0)
Indigenous (%)*				
Non-Indigenous	585 (95)	347 (85)	370 (66)	36 (18)
Indigenous	17 (3)	61 (15)	188 (33)	160 (81)
Missing	11 (2)	1 (0)	5 (1)	1 (1)
History of incarceration (%)*				
No history of incarceration	199 (33)	171 (42)	139 (25)	33 (17)
Yes history of incarceration	395 (64)	205 (50)	350 (62)	158 (80)
Missing	19 (3)	33 (8)	74 (13)	6 (3)
Injected drugs ever (%)*				
No injection drug use	109 (18)	130 (32)	73 (13)	15 (8)
Yes injection drug use	502 (82)	279 (68)	484 (86)	182 (92)
Missing	2 (0)	0 (0)	6 (1)	0 (0)
Injected drugs in the previous 6 months (%)*				
No injection drug use in last 6 months	385 (63)	312 (76)	319 (57)	90 (46)
Yes injection drug use in last 6 months	223 (36)	97 (24)	237 (42)	107 (54)
Missing	5 (1)	0 (0)	7 (1)	0 (0)
Snorted drugs in the previous 6 months (%)				
No snort drugs last 6 months	446 (73)	323 (79)	427 (76)	158 (80)
Yes snort drugs last 6 months	144 (24)	71 (17)	104 (19)	35 (18)
Missing	23 (4)	15 (4)	32 (6)	4 (2)
Sex client ever (%)				
Never sex client	421 (69)	311 (76)	413 (73)	174 (88)
Yes sex client ever	183 (30)	94 (23)	136 (24)	23 (12)
Missing	9 (2)	4 (1)	14 (3)	0 (0)
Sex work ever (%)				
No sex work	417 (68)	328 (80)	340 (60)	151 (77)
Yes sex work	188 (31)	80 (20)	213 (38)	46 (23)
Missing	8 (1)	1 (0)	10 (2)	0 (0)
Sex work in the previous 6 months (%)				
No sex work last 6 months	561 (92)	395 (97)	501 (89)	130 (66)
Yes sex work last 6 months	41 (7)	9 (2)	39 (7)	11 (6)
Missing	11 (2)	5 (1)	23 (4)	56 (28)
Depression (%)				
No depression	419 (68)	170 (42)	224 (40)	88 (45)
Depression	193 (32)	239 (58)	337 (60)	109 (55)
Missing	1 (0)	0 (0)	2 (0)	0 (0)
Psychiatric hospital (%)*				
No psych hospital	466 (76)	310 (76)	406 (72)	154 (78)
Yes psych hospital	127 (21)	99 (24)	149 (27)	43 (22)
Missing	20 (3)	0 (0)	8 (1)	0 (0)
Schizophrenia (%)				
No Schizophrenia	510 (83)	399 (98)	537 (95)	183 (93)
Yes Schizophrenia	22 (4)	7 (2)	14 (3)	13 (7)
Missing	81 (13)	3 (0)	12 (2)	1 (0)
Sexually transmitted disease in the previous 6 months (%)				
No sexually transmitted disease last 6 months	568 (93)	369 (90)	502 (89)	185 (94)
Yes sexually transmitted disease last 6 months	34 (6)	35 (9)	43 (8)	5 (3)
Missing	11 (2)	5 (1)	18 (3)	7 (4)

Variables having an asterisk (*) were included in the final model following the variable selection process.

### Model based index results

**Fig 1:**
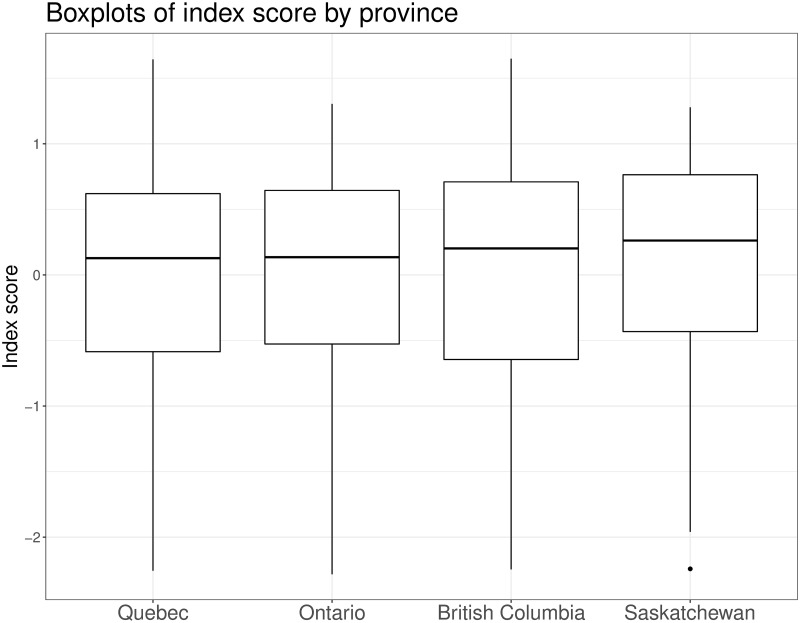
Boxplot of index scores by province. Posterior summaries of the individual scores for each participant presented as boxplots.

The simple model (which assumes α_j_ and β_j_) yielded a WAIC value of 12413.7 (standard error, 116.0) compared to a WAIC of 11960.6 (standard error, 117.0) for the hierarchical model (which assumes α_jk_ and β_jk_). Since the model that allowed parameters to differ across the provinces fit the data better, we only present the results from that model. Boxplots for the individual-level parameter had similar values (Fig 1) suggesting our index has a similar distribution across the four provinces.

**Fig 2:**
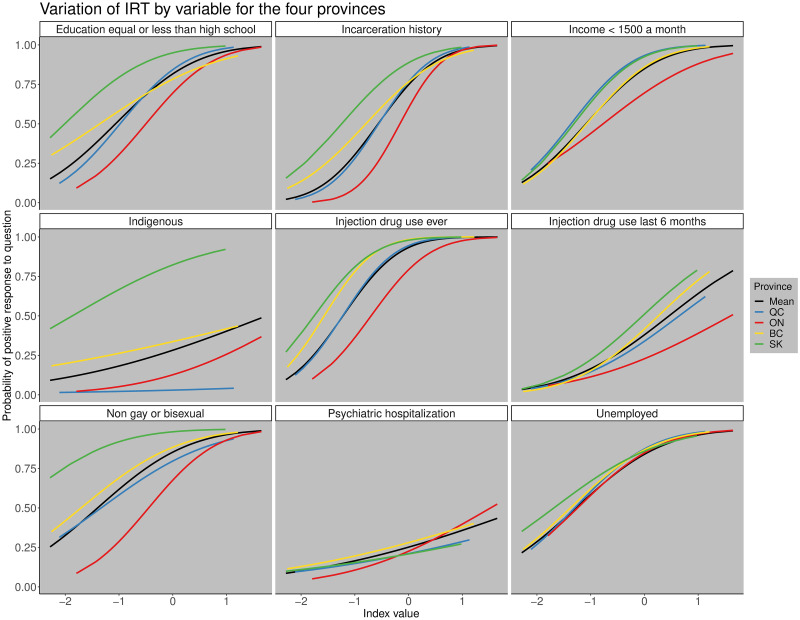
Variation of IRT by variable for the four provinces. Variation of the item characteristic curves for each province by variable. The black line represents the mean value for all the indices, the blue line for Quebec, red line for Ontario, yellow line for British Columbia, and the green line for Saskatchewan. The x−axis shows the value of the estimated deprivation index and the y−axis the probability of responding positively to the question.

However, we found differences in how the response and discriminatory parameters varied by province (ICC curves; Fig 2). The ICC curves show the probability of responding positively to the question (y-axis) based on the value of the deprivation index (x-axis) and the different colored lines show the variation in response by province (Fig 2).

For example, we found that history of incarceration and whether someone identified as gay or bisexual were more important in distinguishing between individuals in the province of Ontario than in the other three provinces. However, there were also some variables that showed little regional variation in the discriminatory parameter like unemployment, which had similar ICCs across the four provinces (Fig 2). The province that differed most from the others was Saskatchewan which had the highest response parameters for people who were Indigenous, do not identify as gay or bisexual, and for education high school or less due to a higher prevalence of those subgroups relative to the other provinces. This reflects that the population of coinfected participants from Saskatchewan differs demographically from other provinces, having more Indigenous people, fewer gay and bisexual males, and a lower proportion of individuals with post-secondary education compared to the rest of the provinces.

### Association of the index and non-attendance at a second visit

Of 1537 eligible participants, 457 (30%) failed to attend a second visit. In the univariable logistic regression, a one-unit increase in the index score was associated with a 17% odds (95% posterior CrI: 2%, 34%), of not attending a second visit. Many of the covariates that make up the index (e.g. non-gay bisexual men who have sex with men (non-GBMSM), and unemployed) were also associated with not attending a visit in univariable analyses, but these estimates were not very precise (Table 2). The multiple regression analysis demonstrates that there is potential for individual components contained in the index to be confounded by, or correlated with, each other if they are included as independent covariates to adjust for deprivation. As expected, including all the covariates in a single multiple regression model reduced precision of individual estimates as the variables are all highly correlated to one another.

**Table 2:** Univariable and multivariable regressions. The point estimates are the mean of the posterior summary and the 95% credible interval of the exponentiated odds ratios.

Variables	Univariable analyses	Multivariable analysis
Index score (per unit)	1.17 [1.02, 1.34]	NA
Education > high school	0.87 [0.68, 1.11]	0.83 [0.62, 1.11]
Incarceration history	0.89 [0.71, 1.13]	0.83 [0.63, 1.10]
Injection drug use ever	0.87 [0.66, 1.17]	0.87 [0.62, 1.24]
Injection drug use in the last 6 month	1.08 [0.86, 1.35]	1.12 [0.88, 1.44]
Non-GBMSM	1.28 [0.98, 1.69]	1.51 [1.11, 2.05]
Unemployed	1.22 [0.92, 1.63]	1.35 [0.98, 1.86]
Income less than $1500 a month	0.91 [0.70, 1.18]	0.84 [0.62, 1.14]
Psychiatric hospitalization	0.96 [0.74, 1.23]	0.98 [0.75, 1.27]
Indigenous	0.95 [0.73, 1.22]	0.96 [0.74, 1.25]

WAIC for the model with the index alone was 1900.7 whereas the WAIC for the multivariable model with all the indicators was 1907.5 indicating that the model using the index alone also had a better goodness of fit.

## Discussion

We developed an individual-level index incorporating social, material, and lifestyle components for Canadians who are coinfected with HIV/HCV. We examined how the characteristics of individuals differed for different values of the estimated index. Individual components of the model varied by province—reflecting local HIV-HCV epidemiology–while values of the index were distributed similarly across the country. High values of this index identified participants with more vulnerable profiles. We illustrated how the index could be used to evaluate an outcome that should be affected by deprivation–higher values of the index were associated with lack of attendance at a second cohort visit.

The index has several advantages over the most commonly used deprivation index, the Pampalon index [6]. Unlike the Pampalon index, an area based index, that values of our index represents an individual-level measure of deprivation compared with assigning everyone in the same census area the same value of deprivation [31]. This allows us to assign an individual a deprivation value that is reflective of their personal characteristics [32]. Additionally, our index included variables which are particularly relevant for deprivation in HIV-HCV coinfected individuals and have not been used in any previous deprivation indices to our knowledge such as incarceration history [3, 4, 10, 33–38].

As a single summary measure of deprivation, the index offers value in that it captures the effects of multiple variables that contribute to deprivation simultaneously, preserving degrees of freedom and thus offering improved precision. As a tool for adjusting for deprivation in future studies, our index summarizes the shared variation among the variables eliminating the need for including multiple covariates to control for deprivation and therefore is more parsimonious, which is beneficial. We illustrate this when evaluating a potentially relevant outcome, disengagement from care. The model with the index alone provided a much lower WAIC compared to the multivariable model with all variables contained in the index assuming a difference in WAIC of greater than 5 between two models is considered as a substantial difference [26]. Additionally, the individual estimates from the model that contained all the covariates led to wider ranges of the 95% posterior credible intervals, indicating that the posterior variance of the coefficients was greater when compared to the coefficients from the univariate model counterparts. This is probably because these covariates are correlated (Table 2). Our index naturally accounts for this correlation among these covariates, summarizing the characteristics of an individual into a single number. This allows for us to be able to compare individuals on a continuous range of numbers based off a mathematical transformation of 9 dichotomous variables.

### Limitations

The main limitation of our study was that the items selected for inclusion in the index were partly subjective. Experts may differ on what variables they believe should be included in the model. In our model, we included the 9 variables using a variable selection process, which considered the peer-reviewed literature, statistical associations amongst the candidate variables and *a priori* expert opinion. Another limitation is that the index is based exclusively on the data collected in our cohort, therefore, our score may not generalize well to coinfected individuals in other settings. However, the CCC is a very diverse cohort and is representative of a wide range of individuals across Canada in multiple provinces [11]. Additionally, this index cannot be used directly in another cohort. The process would need to be replicated by fitting the IRT model to the other cohort’s data. However, our index identified distinct characteristics that differed amongst the four provinces reflecting local epidemiology, despite their diverse socio-demographics, it is reasonable to assume that if we were to refit this model in another high-income country similar to Canada, like Australia, that it will be able to be flexible in accounting for group differences that may exist, provided that the same variables are available and data are robust. We did not impute data for missing variables as missing data were rare. We acknowledge that using complete cases could potentially introduce bias if data were not missing at random. From a Bayesian point of view, a model for the missing mechanism could be jointly fitted with the IRT model and the missing values could have been estimated in situations with higher rates of missing data.

Furthermore, we dichotomized the variables that were included in the model and this could lead to a loss of information. Although there are more levels in many of our variables we decided to dichotomize them due to concerns about sample size in small sub categories. Moreover, a strict monotonically increasing order is required for all variables in the model. When adding more categories, we need to make sure that there is a clear hierarchy among them, which is not possible for some variables such as Indigenous status, or non-GBMSM, which would need to have more than 2 categories. Finally, the outcome that we used was non-attendance of a second cohort visit, and acknowledge that this may not accurately capture disengagement from care as individuals could attend a subsequently scheduled appointment. Gaps in attendance in care is common in more marginalized populations who have difficulty accessing the healthcare system for a variety of reasons and may only attend erratically.

## Conclusions

We developed and estimated a novel individual-level index that measures deprivation in a national representative cohort of HIV/HCV coinfected Canadians. Due to the hierarchical nature of the model, variables were able to contribute differently depending on the province each individual was from and reflected the local epidemiology. The proposed index is a valuable tool to understand the profile of any individual that is part of the CCC study. In particular, our proposed approach is able to provide an estimate for the index of new participants, potentially allowing health workers to better understand the profile of a new individual with respect to others in the study. If validated in other settings, it may be a valuable tool for studying health outcomes in HIV-HCV coinfection in observational studies.

## Supporting information

S1 File Selection criteria output. This contains supplemental output for the selection criteria that were used to select variables into the final model.
(PDF)
