# Supplementary material for: Estimating an individual-level deprivation index for HIV/HCV coinfected persons in Canada
Source: PLoS One. 2021 Apr 19;16(4):e0249836. doi: 10.1371/journal.pone.0249836 (PMC8055038; doi:10.1371/journal.pone.0249836)
Supplement: S1 File — This contains supplemental output for the selection criteria that were used to select variables into the final model. (PDF) [file pone.0249836.s001.pdf]

Estimating an individual-level deprivation index for HIV/HCV co-infected persons in Canada: Appendix 1

Apendix 1 selection criteria results

This is appendix 1 for the manuscript titled, "Estimating an individual-level deprivation index for HIV/HCV co-infected persons in Canada". In this appendix we present the graphical output of the selectrion criteria that is described in the methods of the article. In this appendix we present two heat-maps of chi-squared p-values to show all pair-wise statistical associations within the two different groups of variables. Additionally, we present the results of multiple joint correspondance analyses (MJCA) for the two groups of variables.

List of figures in appendix 1

`* Figure 1: Heatmap of p-values from pairwise chi-squared tests of the Pampalon variables`

`* Figure 2: Heatmap of all p-values from pairwise chi-squared tests of the literature variable`

`* Figure 3: Multiple joint correspondence analysis of the Pampalon variable`

`* Figure 4: Multiple joint correspondence analysis of the Literature variable`

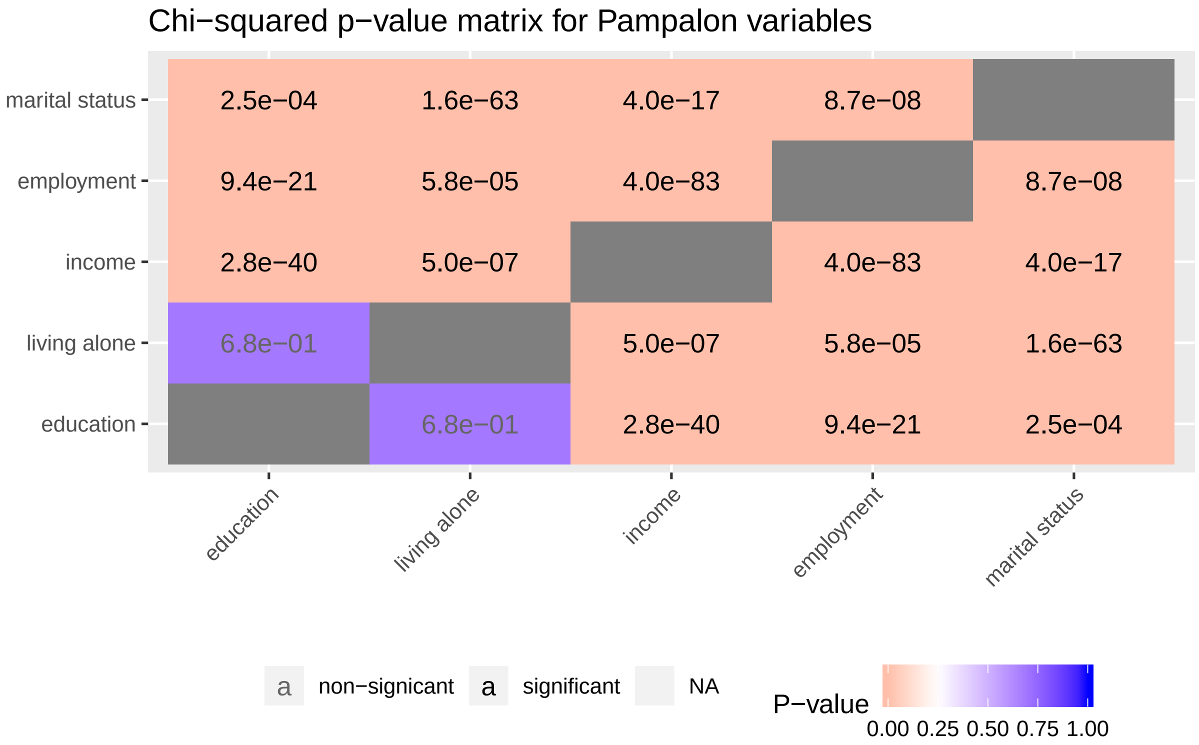

Figure 1: Heatmap of all p-values from pairwise chi-squared tests of the Pampalon variable. Text values in white are considered statistically significant at an alpha = 0.05 and values in black text are non-significant at an alpha = 0.05.

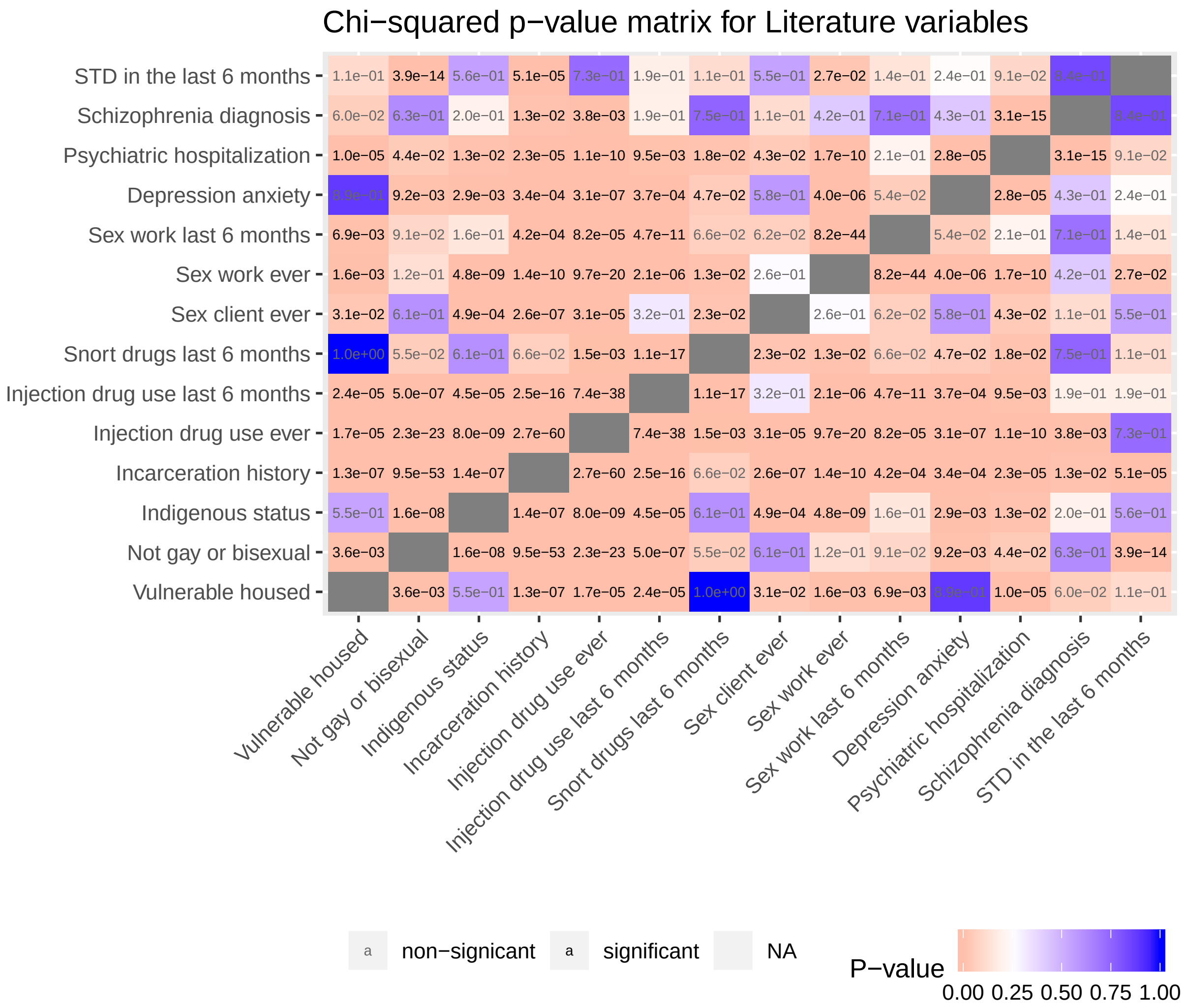

Figure 2: Heatmap of all p-values from pairwise chi-squared tests of the literature variable. Text values in white are considered statistically significant at an alpha = 0.05 and values in black text are non-significant at an alpha = 0.05.

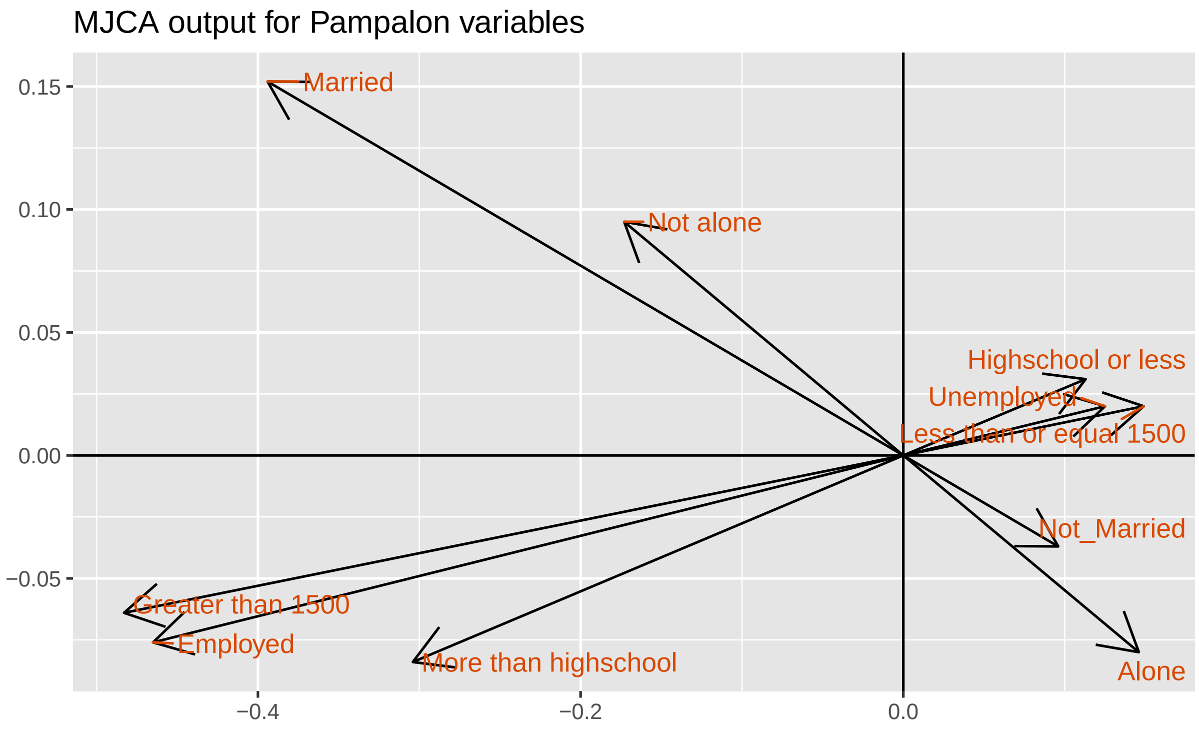

Figure 3: Multiple joint correspondence analysis of the Pampalon variables. We can see from the Pampalon variables that there is an association between variables along the major x-axis.

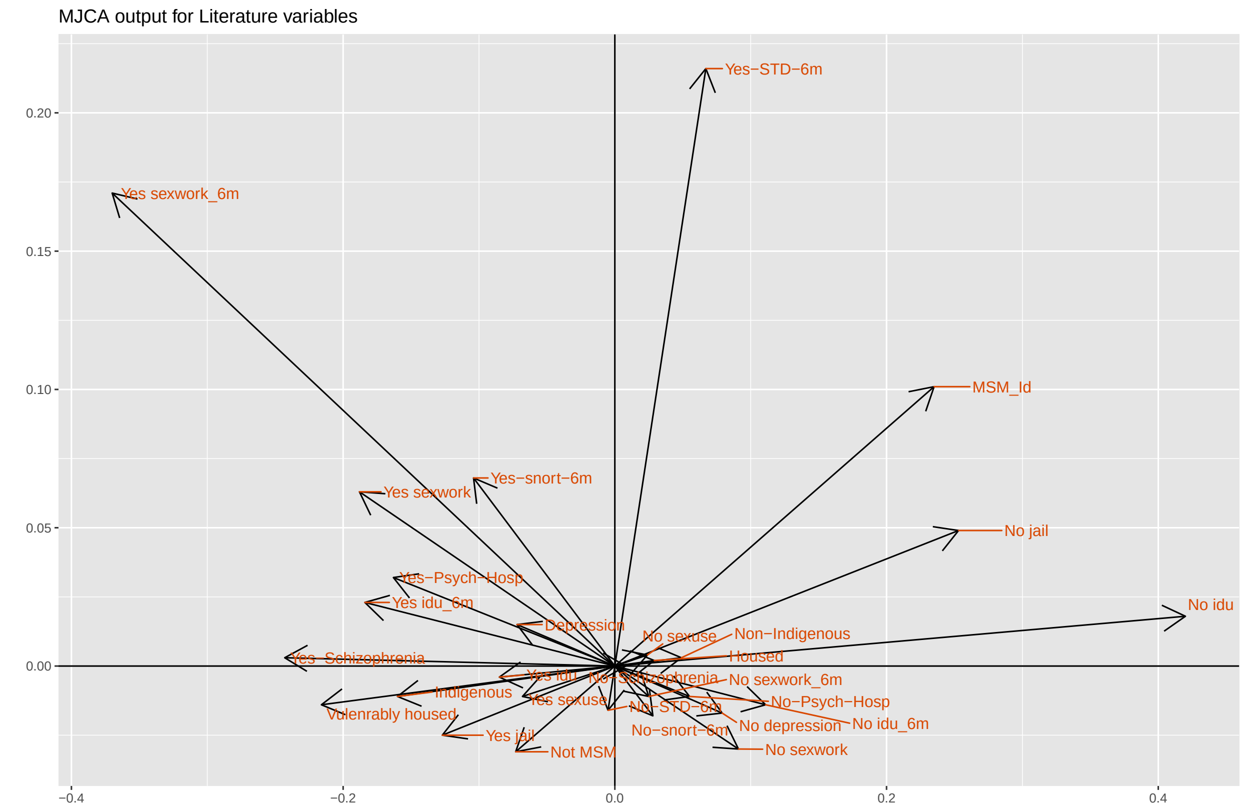

Figure 4: Multiple joint correspondence analysis of the literature variables. We can see that there is an association of variables of interest like injection drug use, MSM, and psychiatric hospitalization that map well will the major x-axis.
